# Comparative Study of Predominant Phytochemical Compounds and Proapoptotic Potential of Broccoli Sprouts and Florets

**DOI:** 10.1007/s11130-018-0665-2

**Published:** 2018-04-19

**Authors:** Paweł Paśko, Małgorzata Tyszka-Czochara, Agnieszka Galanty, Joanna Gdula-Argasińska, Paweł Żmudzki, Henryk Bartoń, Paweł Zagrodzki, Shela Gorinstein

**Affiliations:** 1 Department of Food Chemistry and Nutrition, Medical College, Jagiellonian University, Medyczna 9, 30-688 Kraków, Poland; 2 Department of Pharmacognosy, Medical College, Jagiellonian University, 30-688 Kraków, Poland; 3 Department of Radioligands, Medical College, Jagiellonian University, 30-688 Kraków, Poland; 4 Department of Medicinal Chemistry, Medical College, Jagiellonian University, 30-688 Kraków, Poland; 5 Institute for Drug Research, School of Pharmacy, Hadassah Medical School, The Hebrew University, 91120 Jerusalem, Israel

**Keywords:** Broccoli sprouts and florets, Freeze-dried products, Sulforaphane, Fatty acids, Apoptosis

## Abstract

The study compares lyophilized broccoli sprouts and florets in terms of their chemical composition, cytotoxic and proapoptotic potential against hepatocellular carcinoma HepG2, colorectal cancer SW480, and skin fibroblast BJ cells. Sinapic and isochlorogenic acids were predominant phenolics in the sprouts and florets, respectively. The amount of sulforaphane in the sprouts was significantly higher vs. florets. Oleic and linoleic acids dominated in the sprouts, while caproic, stearic and oleic acids in the florets. Broccoli sprouts were selectively cytotoxic on HepG2 and SW480 cells, with proapoptotic effect for the latter, while the florets were less selective, but more active, with profound proapoptotic effect for HepG2 cells (77.4%). Thus, lyophilized broccoli sprouts may be effectively used in dietary chemoprevention.

**Electronic supplementary material:**

The online version of this article (10.1007/s11130-018-0665-2) contains supplementary material, which is available to authorized users.

## Introduction

Broccoli (*Brassica oleracea* L. var. *italica, Brassicacae*) are a good source of isothiocyanates, with most important sulforaphane (SF), known for its chemopreventive properties [1], but are also rich in vitamins (C, K, β-carotene), dietary fibre, polyphenols and fatty acids [2], with considerable health beneficial effects [3]. Broccoli marketable sprouts, florets, flour, fiber, flakes, powder, crisps, etc. are under vast interest nowadays, due to their preventive role in non-communicable diseases, like hypertension, atherosclerosis [2, 4] or cancer (lung, breast, prostate and pancreatic) [1]. Consumption of *Brassica* vegetables may decrease the risk of gastric cardia and esophageal adenocarcinomas [5], but also colon and colorectal cancers [6] in humans. The so far described phytochemical composition of broccoli concerns mostly fresh material, while many studies discuss the influence of processing methods (cutting, boiling, steaming, etc.) on the loss of active substances, especially SF [7, 8]. Moreover, fresh broccoli are difficult to store for more than a few weeks and, except from the storage in argon or helium atmospheres [9], the data on the alternative methods of preservation is scarce. The studies on the impact of storage conditions on phytochemical content of fresh and lyophilized broccoli products are still limited. Therefore, the aim of our study was qualitative and quantitative analysis of lyophilized broccoli sprouts and florets, focusing on polyphenols (flavonoids, phenolic acids), SF, and fatty acids. Moreover, we also studied broccoli products’ effect on the viability and apoptosis induction in human cancer and normal cells, to determine not only the cytotoxic potential, but also selectivity of the tested samples and the mechanism of cell death.

## Material and Method

### Plant material and Freeze - Drying Method

Broccoli seeds (Brassica oleracea L. var. italica) were obtained commercially [10], detailed information about the material and freeze - drying method is described in supplementary material.

### Extracts Preparation

Extraction process was described in details in supplementary material. SF and lipids were extracted according to [11, 12].

### HPLC Determination of Flavonoids and Phenolic Acids

The analysis was performed according to [13] and is described in supplementary material.

### UPLC/MS Determination of Sulforaphane Content

The analysis was performed according to [14] and is described in supplementary material.

### GC Determination of Fatty Acids Profile

The analysis was performed according to [12] and is described in supplementary material.

### Cytotoxicity Assay, Apoptosis and Necrosis Analysis

The analysis was performed according to [15–17] and is described in supplementary material.

### Statistical Analysis

Statistical differences were determined using Student’s *t*-test. *P* < 0.05 was considered to be statistically significant. The hierarchical principal component analysis was used to reveal the correlation structure between the investigated parameters [14, 18] and is described in supplementary material.

## Results and Discussion

### Phenolic Acids and Flavonoids

Flavonoids and phenolic acids are phytochemicals widespread in brassica vegetables [19]. Qualitative HPLC analysis of broccoli sprouts revealed chlorogenic, *p*-coumaric, ferulic, gentisic and sinapic acids, and also robinin, with traces of myricetin, luteolin, quercetin and apigenin. In broccoli florets only caffeic, isochlorogenic and sinapic acids and also traces of myricetin were detected. Although some peaks on HPLC chromatograms remained unidentified, we decided to determine and compare the content of the recognized polyphenols in both extracts by means of quantitative HPLC analysis. The results (Table 1) revealed vast differences in the amounts of the phenolic acids of the tested broccoli sprouts and florets extracts. Sinapic, gentisic, and ferulic acids predominated in broccoli sprouts, with chlorogenic and *p*-coumaric acids in smaller amounts. In contrast, isochlorogenic acid, not detected in sprouts, was present in the highest amount in broccoli florets, accompanied with sinapic and caffeic acids. A range of phenolic acids have been identified so far in broccoli sprouts including chlorogenic, gallic, sinapic, ferulic, benzoic, vanillic, protocatechuic or *p*-coumaric acid [20–23], while neochlorogenic and chlorogenic acids, together with sinapic and ferulic acids derivatives, were detected in the florets. The results of Pająk et al. [20] indicated similar qualitative phenolic acids profile, but with quantitative differences: ferulic (7.66 mg/100 g dw), *p*-coumaric (2.04 mg/100 g dw) and chlorogenic (11.49 mg/100 g dw) acids were in lower, while sinapic acid (548 mg/100 g dw) was in higher amounts, in comparison to our results. For broccoli florets, our results are similar to those by Fernández-León et al. [21] for sinapic acid in some Monaco cultivars. Our results indicated robinin, a kaempferol glucoside, to be the only flavonoid identified in broccoli sprouts. Similar results were reported by Pająk et al. [20] and Gawlik-Dziki et al. [22], indicating kaempferol as the major compound in broccoli sprouts after hydrolysis. Other authors identified also luteolin, apigenin, quercetin, and kaempferol, contributing altogether to 2.77–14.66 mg/100 g dw [20, 21].

**Table 1:** Content of phenolic acids, flavonoids, sulforaphane and fatty acids profile of dry broccoli sprouts and florets

	Broccoli sprouts	Broccoli florets
	Phenolic acids [mg/100 g dw] (n = 3)	
Caffeic acid	ND	1.55 ± 0.05
Chlorogenic acid	37.26 ± 0.60	ND
Iso-chlorogenic acid	ND	59.85 ± 2.56
*p*-Coumaric acid	27.75 ± 0.70	ND
Ferulic acid	73.85 ± 4.50	ND
Gentisic acid	80.80 ± 4.79	ND
Sinapic acid	140.53 ± 3.17^*^	3.43 ± 0.15^*^
	Flavonoids [mg/100 g dw] (*n* = 3)	
Robinin	1.64 ± 0.10	ND
	Sulforaphane [mg/100 g dw] (n = 3)	
	113.33 ± 12.58^*^	46.46 ± 7.50^*^
	Fatty acids profile [%] (n = 3)	
Saturated acids	10.5	77
C6:0 caproic acid	0.3 ± 0.1*	34.9 ± 2.9*
C10:0 capric acid	ND	9.2 ± 0.5
C14:0 myristic acid	0.3 ± 0.1	ND
C16:0 palmitic acid	5.7 ± 0.1*	12.0 ± 1.0*
C18:0 stearic acid	2.8 ± 0.1*	15.9 ± 1.1*
C24:0 lignoceric acid	1.4 ± 0.0*	5.0 ± 0.3*
Unsaturated acids	88.6	23
C18:1 oleic acid (n-9)	45.5 ± 0.5*	12.4 ± 0.5*
C18:2 linoleic acid (n-6)	20.8 ± 0.4*	3.8 ± 0.6*
C18:3 alpha-linolenic acid(n-3)	17.0 ± 0.1	ND
C20:1 eicosenoic acid (n-9)	0.8 ± 0.0*	4.8 ± 0.3*
C22:1 erucic acid (n-9)	0.5 ± 0.0*	2.0 ± 0.1*
C22:2 docosadienoic acid (n-6)	4.0 ± 0.0	ND

ND – not detected

^*^p < 0.05

### Sulforaphane Content

The content of SF is given in Table 1. Broccoli sprouts were significantly richer in SF than the florets, and the results are consistent with Nakagawa et al. [23], who indicated 10 times higher amount of SF in the sprouts in comparison to mature plants. Our results are also similar to López-Cervantes et al. [24], while the results of Moon et al. [2] for 6 days sprouts (222.6 mg/kg fw) and Nakagawa et al. [23] for sprouts from the market (1153 mg/100 g dw) were higher than ours. Such differences may be due to the plant variety or growing and sprouting condition. Our results for SF content in broccoli florets comply with Nakagawa et al. [23] for broccoli from Japan (69.1–171.3 mg/100 g dw) and Campas-Baypoli et al. [25], for lyophilized florets from Mexico (31–45.4 mg/100 g dw). Many studies, including our results, proved that broccoli sprouts are better source of SF than the florets [23, 24], which implies their use as dietary prevention of different health problems.

### Fatty Acid Profile

Fatty acid analysis was performed by means of gas chromatography. Preliminary qualitative GC analysis of broccoli sprouts extracts indicated the presence of caproic, myristic, palmitic, stearic, arachidic, lignoceric, palmitooleic, oleic, linoleic, α-linolenic, eicosenoic, erucic and docosadienoic acids, while in broccoli florets caproic, capric, palmitic, stearic, lignoceric, oleic, linoleic, eicosenoic and erucic acids were detected. For detailed comparison of the tested materials, the percentage of saturated (SFAs) and unsaturated (UFAs) fatty acids was further determined by GC. SFAs in broccoli sprouts and florets consisted 10.5 and 77% of total pool of fatty acids, respectively, with palmitic (5.7%) and stearic (2.8%) acids dominating in sprouts, and caproic (34.9%), stearic (15.9%) and palmitic (12%) acids in florets (Table 1). Qualitative fatty acids profile in both evaluated materials was similar to Ahmed et al. [26] for *Brassica juncea* and López-Cervantes et al. [24] for broccoli sprouts. We found the relative content of UFAs in the analyzed broccoli sprouts to be 88%, with the predominant oleic acid (45.5%) but linoleic (20.8%) and α-linolenic acids (17.0%) on similar level. In broccoli florets oleic acid was also the main UFA (12.4%), but in significantly lower amount in comparison to the sprouts. The results of López-Cervantes et al. [24] indicated linoleic (26.31–31.88%) and α-linolenic acids (21.34–28.69%), with lower amount of oleic acid (5.17–11.70%) in broccoli sprouts. What is particularly important, our results revealed significantly higher total UFAs content in the sprouts in comparison to the florets, with very low amounts of harmful erucic [27] acid in sprouts (0.5%) and florets (2%), in comparison to the broccoli seeds (38% - data not shown). Another study of three South Korea broccoli cultivars revealed much lower content of total SFAs, and higher amount of UFAs, with the predominance of palmitic and linolenic acids, in the florets, when compared to our results [28].

### Cytotoxic Activity

**Fig. 1:**
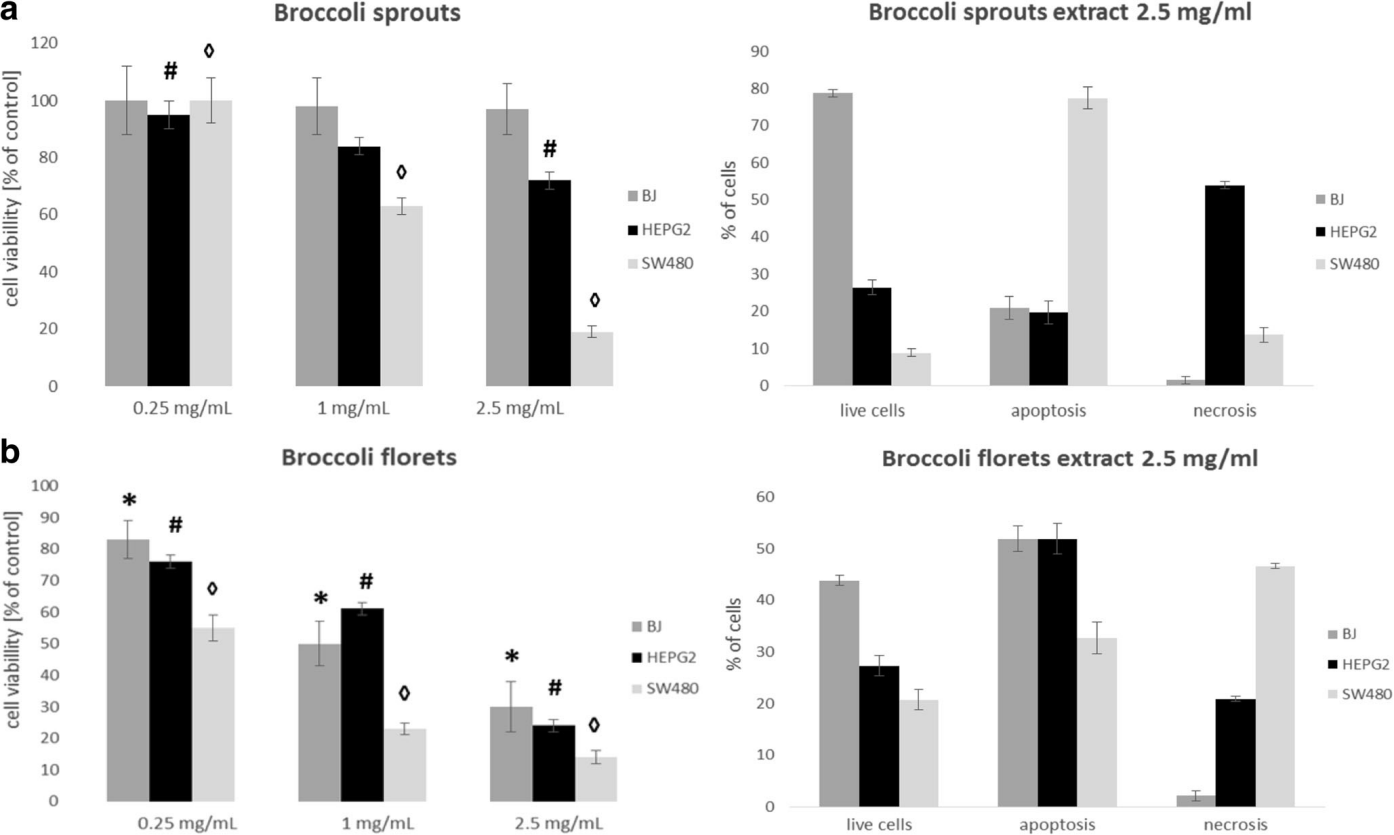
Cytotoxic activity of methanol broccoli sprouts (**a**) and florets (**b**) extract on BJ, SW480, HepG2 cells (left two diagrams) and apoptosis/necrosis induction of the 2.5 mg/mL extracts (right two diagrams). Cells were cultured in the presence (0.25–2.5mg/mL) of dry broccoli extracts. Cell viability was expressed as % of control (untreated) cells. Values represents means ±SEM; each experiment was done in triplicate. Means with the same symbols on each subdiagram differ significantly (*p* < 0.05)

Cytotoxic impact of the tested broccoli products was performed by MTT assay against cancer HepG2 and SW480, but also normal BJ cells, to determine the potential selectivity. The tested samples varied in their influence on the examined cell lines, in dose dependent manner. Both broccoli sprouts and florets caused significant decrease in SW480 and HepG2 cancer cells viability (Fig. 1), with more profound effect for the former and less susceptibility of the latter. The cytotoxic effect of broccoli florets was stronger for both cancer cell lines in comparison to the sprouts. It is worth to note that the latter were also more selective against cancer cells, as opposed to the florets, affecting also normal fibroblasts. Such effects may result from the differences in phytochemical content of both products. Broccoli sprouts with the predominance of polyphenolics and SF, known from their antioxidant and protective effects [1, 3], actually did not affect normal fibroblasts, while the opposite results were observed for the florets, with lower content of the mentioned protective compounds. Stronger cytotoxic effect against cancer cells by broccoli florets, especially in higher concentration, may result from the presence of SFAs, and the effect was also observed by Lima et al. [29]. Similar experiment on cytotoxic activity of broccolini (*Brassica oleracea Italica x Alboglabra*), a hybrid between broccoli and Gai Lan, on four human cancer cell lines (SW480, HepG2, Hela, and A549) was performed by Wang & Zhang [30] and the results showed a dose-dependent antiproliferative properties of broccolini leaves extract. Cytotoxic activity of broccoli sprouts was previously studied on human leukemic cell line (HL-60), with no significant effects on proliferation or viability [31] and on two murine prostate cancer cell lines, AT2 and Mat-Ly-Lu, with interesting effect, correlated with antioxidant activity [22]. Broccoli florets extracts were tested on human ovary (OVCAR-5), breast (MCF-7), colon (Colo-205) and prostate (PC-3) cancer cells [23] and significant effect on human lung cancer cells A-549 was also described [32]. All the results indicate that broccoli sprouts and florets may be considered as potent cancer chemopreventive agents.

### Apoptosis and Necrosis

To further investigate the impact of the tested broccoli sprouts and florets on the cells, their proapoptotic potential was evaluated by means of flow cytometry (Fig. 1). Strong cytotoxic effect of broccoli florets on SW480 cells, described above, proved to be due to necrosis (46.6%) rather than apoptosis (32.7%), while the opposite effect was observed for HepG2 (20.9 and 51.9%, respectively) and for normal fibroblasts (2.1 and 51.9%, respectively). On the contrary, the tested broccoli sprouts, with minor cytotoxic effect, stimulated apoptosis in SW480 (77.4%) and necrosis (50%) in HepG2 cancer cells, and their slight impact on normal fibroblasts was due to proapoptotic effect (20.9%). Our findings for broccoli sprouts were also proved for SW480 cells by Wang & Zhang [30] and Hudson et al. [33], but also for human bladder carcinoma cells [34]. Our study is probably the first to demonstrate significant differences between mechanism of cell death between dry broccoli sprouts and florets. Such strong effect of broccoli sprouts on SW480 cells in comparison to florets may expand dietetic recommendation for chemoprevention of colorectal cancer, though further *in vitro* and *in vivo* studies are needed. The statistically significant HPCA model of two significant components was derived. Table 1S shows the details of PCA models for blocks X1 and X2 as well as for overall HPCA model. Block X1 was described completely by only one principal component which accounts for 99.2% of the variation in this block. All variables in this block had similar absolute values of their loadings (around 0.35), but for C16:0, C18:0, C24:0, C20:1 and C22:1 with positive, while for C18:1 and C18:2 with negative signs, which therefore indicates inverse correlation between the last two and the rest of fatty acids as a whole group. Figure 1S shows HPCA final plot for the first two components. The first principal component in this model had positive loadings for APOPT-SW480, SF, sinapic acid, and first principal component from the block X2, which all were interrelated with correlation weighs of ca. 0.13–0.14, and simultaneously correlated negatively with APOPT-BJ, APOPT-HEPG2 and principal component from the block X1 (again with correlation weighs with absolute values in the same range as above). Being in one cluster of variables (APOPT-BJ, APOPT-HEPG2 and principal component from the block X1) means also that APOPT-BJ and APOPT-HEPG2 were positively interrelated with fatty acids C16:0, C18:0, C24:0, C20:1 and C22:1, and negatively with C18:1 and C18:2. Such relationships suggest that sinapic acid, SF and two fatty acids (C18:1 and C18:2) inhibit apoptosis of BJ and HepG2, while other fatty acids promote it. Second principal component in HPCA was solely loaded by second principal component from X2 block predominantly influenced by NECRO-BJ and NECRO-HEPG2. The separation between BS and BF samples in HPCA model was evident in direction determined only by the first principal component of HPCA model. Considering this result, BS samples were characterized by apparently higher values of sinapic acid, SF, C18:1, C18:2, MTT-BJ-2.5, and MTT-HEPG2–2.5, and lower values of APOPT-BJ, APOPT-HEPG2, C16:0, C18:0, C24:0, C20:1 and C22:1 as compared with BF samples. This is consistent with results delivered by *t*-test analysis.

## Conclusions

The results of our study provide new and more precise insights into phytochemical content and potential health benefits of broccoli sprouts and florets. Lyophilized sprouts and florets do substantially differ in their polyphenolic, sulforaphane and fatty acid profile and amounts. Moreover, the examined sprouts exhibited significant, selective cytotoxic and proapoptotic effect against colorectal cancer, without a toxic impact on normal cells. Such properties may be a strong argument for recommending lyophilized sprouts as functional broccoli product, with chemopreventive potential against large intestine tumor.

## Electronic Supplementary Material

ESM 1 (DOCX 36 kb)
