# Supplementary material for: Comparative Study of Predominant Phytochemical Compounds and Proapoptotic Potential of Broccoli Sprouts and Florets
Source: Plant Foods Hum Nutr. 2018 Apr 19;73(2):95–100. doi: 10.1007/s11130-018-0665-2 (PMC5956025; doi:10.1007/s11130-018-0665-2)
Supplement: Supplementary file 1 — (DOCX 36 kb) [file 11130_2018_665_MOESM1_ESM.docx]

Supplementary material

1.Article title: Comparative study of predominant phytochemical compounds and proapoptotic potential of freeze-dried broccoli sprouts and florets

2.Journal name: *Plant Foods for Human Nutrition*

3.Corresponding author name: Paweł Paśko

4.Corresponding author affiliation: Department of Food Chemistry and Nutrition, Medical College, Jagiellonian University, Medyczna 9, 30-688 Kraków, Poland

5.Corresponding author E-mail address: [paskopaw@poczta.fm](mailto:paskopaw@poczta.fm)

**Material and Methods**

***Plant material***

Broccoli seeds (*Brassica oleracea L. var. italica*) were obtained commercially from plants harvested in northern Poland (Gdańsk). Sprouts were grown for 4 days after seeding at the fixed temperature of 20±2ºC. They were watered every day and harvested in natural light conditions. Broccoli florets were harvested in southern Poland (Krościenko n/Dunajcem – GPS; N49 26'26.88'' E20 25'42.54'') in October 2015.

***Freeze - Drying method***

Samples of raw material were frozen at −20 °C for 48 h before freeze drying process. The plant material was freeze dried using an VEB Hochvakuum (Germany), type TG 15.4 freeze dryer, comprising of a drying chamber, a cold trap, a vacuum pump, and measurement and control system. The freeze dryer was also equipped with a heating system. The process of drying was continued at 30 and 40 °C with a constant pressure in the drying chamber until the mass of the sample reached a moisture of about 70 g H_2_O/kg fresh weight.

***Extracts preparation***

Samples of lyophilized sprouts and florets (5 g) were grinded and extracted with methanol (50 mL) for 3 hours in a Soxhlet apparatus. The obtained methanol extracts were decanted, centrifuged, stored in darkness in a freezer at –20°C and then were used for HPLC analysis of polyphenolic compounds. For cell viability and proapoptotic assays the extracts obtained from Soxhlet extraction were evaporated under reduced pressure and the dry residues were dissolved in DMSO. All solutions for cytotoxic assay were kept at -20°C for less than a week. Lipids were extracted from broccoli sprouts and florets with chloroform: methanol solution (2:1 v/v), with the addition of butylated hydroxytoluene at 0.005% to prevent unsaturated fatty acids peroxidation.

***HPLC determination of flavonoids and phenolic acids***

The identification and quantification of flavonoids and phenolic acids was performed with Dionex HPLC system with PDA 100 UV-VIS detector, Hypersil Gold (C-18) column (5 μm, 250 × 4.6 mm, Thermo EC) mobile phase 1% formic acid in water (A) and acetonitrile (B) in gradient mode 5–60% B in 60 min, at a flow-rate of 1 mL/min. Identification of the compounds was done by comparing their retention time with those of the standards. Quantification of the predominant compounds was carried out by measuring the peak area with regards to the appropriate standard curve prepared for the range of concentrations from 0.0625 to 1 mg/mL. All analyses were performed in triplicate and the mean value was expressed in mg/100 g dry weight (dw).

***UPLC/MS determination of sulforaphane content***

The UPLC-MS/MS system consisted of Waters ACQUITY^®^ UPLC^®^ (Waters Corporation, Milford, MA, USA) coupled to Waters TQD mass spectrometer (electrospray ionization mode ESI-tandem quadrupole). Chromatographic separations were carried out using the Acquity UPLC BEH (bridged ethyl hybrid) C18 column, 2.1 × 100 mm, and 1.7 µm particle size. The column was maintained at 40°C, and eluted under the following conditions: 100% of eluent A over 2 min, linear gradient elution from 100% to 50% of eluent A over 3 min, linear gradient from 50% to 0% of eluent B over 2 min, at a flow rate of 0.3 mL/min. Eluent A: water/formic acid (0.1%, v/v); eluent B: acetonitrile/formic acid (0.1%, v/v). Total volume of 10 μL of each sample was injected in triplicate. All analyses were performed in triplicate and the average value was expressed as mg/100 g dw.

***GC determination of fatty acids profile***

Agilent 6890N gas chromatograph, capillary column DB-23 (50% Cyanopropyl-methylpolysiloxane 60 m, ID 0.25 mm, film 0.25 µm), FID detector; carried gas was helium (He), used at a rate of 0.5 mL/min. Fatty acid methyl esters (FAME) were synthesized with 20% boron trifluoride in methanol at 60°C. The analysis of FAME was performed with gas chromatography. For all the fatty acids the mean value of three replicates was expressed as the percentage of the total fatty acids pool.

***Cell analysis***

*Cell culture conditions*

Human cell lines were derived from the American Type Cell Culture collection, ATCC (LGC Standards-ATCC, Teddington, Great Britain). ATCC designations were as follows: BJ, normal adherent human skin fibroblasts, CRL-2522; HepG2, hepatocellular carcinoma, HB-8065 and SW480, Duke’s type B colorectal adenocarcinoma, CCL-228. The cells were grown as monolayer cultures in Eagle’s Minimum Essential Medium, EMEM (the skin fibroblasts BJ and HEP G2 cells) or in Dulbecco’s Modified Eagle’s Medium, DMEM (SW 480 cells) (Sigma-Aldrich, Seelze, Germany). Media were supplemented with 10% Fetal Bovine Serum (PAA Laboratories GmbH, Austria) and 1% antibiotic (100 IU/ml penicillin, 0.1 mg/mL streptomycin) (Gibco Laboratories, NY, USA). Trypsin 0.05% EDTA solution was used to detach adherent cultures (Gibco Laboratories, NY, USA). Cells viability during the culture was verified with Trypan Blue Exclusion Dye Test and automatic cell counter, and the percentage of living cells during the culture was 95–98%.

*Cytotoxicity assay*

In order to determine cytotoxic action of tested broccoli sprouts and florets, the MTT assay was performed. The cells (1x10^5^ cells/well) were incubated with different concentrations of broccoli sprouts and florets dry methanolic extracts (0.25 to 2.5 mg/mL) for 24 hours, then the media were changed for new ones with the addition of 5 mg/mL MTT. Results were expressed as a number of metabolically active cells related to the control cells. All analyses were performed in triplicate and were expressed as means±SD.

*Apoptosis and necrosis analysis.*

The cells were incubated with appropriate concentrations of the tested extracts for 24 h. Commercially available kit (Biotium, Fremont, CA, USA) with fluorescent dyes Annexin-V (Ann-V, excitation/emission 490/515 nm) and Ethidium homodimer (EthD-III, excitation/emission 528/617 nm) was used for the assay, according to manufacurer’s protocol. Cell suspensions were gated according to forward (FSC), side scatter (SSC), and appropriate fluorescence parameters. The living cells were defined as negative for Ann-V and EthD-III, the apoptotic cells consisted of Ann-V positive/EthD-III negative cells (early apoptosis) and Ann-V/EthD-III positive cells (late apoptosis); the necrotic cells were Ann-V negative and EthD-III. The results were given as the percentage of apoptotic or necrotic cells of the total counted cells.

***Statistical analysis***

The assay was conducted with three replicates for each treatment. Data were expressed as means±SD values. Statistical differences were determined using Student's t-test.P<0.05 was considered to be statistically significant. The hierarchical principal component analysis (HPCA) was used to reveal the correlation structure between the investigated parameters and to improve the interpretability of results. In this method new variables are created by blocking original data into meaningful subgroups for which PCA models are built. New variables obtained as principal components for such subgroups are then used in superior (hierarchical) PCA model. In our work block X1 consisted of concentrations of eight fatty acids: C6:0, C16:0, C18:0, C24:0, C18:1, C18:2, C20:1, C22:1. Block X2 contained following parameters: MTT, percent of necrotic cells in all evaluated cell lines for the highest extract concentration (2.5 mg/mL) (MTT-BJ-2.5, MTT-HEPG2-2.5, MTT-SW480-2.5, NECRO-BJ, NECRO-HEPG2, and NECRO-SW480). Remaining parameters outside any block were: sinapic acid, APOPT-BJ, APOPT-HEPG2, APOPT-SW480, and sulforaphane. The association between two parameters was quantified by calculating their correlation weights. The calculations of PCA model were carried out with the package SIMCA-P v. 9 (Umetrics, Umeå, Sweden). The correlation weights were calculated with software delivered by MP System Co. (Chrzanów, Poland).

**Supplementary Table 1.** The percentage of explained variation of two blocks (X1 and X2) within HPCA and for the whole model.

| Component | Percentages of explained variation | Cumulative percentages of explained variation | Eigenvalues |
| --- | --- | --- | --- |
| PC1 in X1 | 99.2 | 99.2 | 5.95 |
| PC1 in X2 | 90.4 | 90.4 | 5.43 |
| PC2 in X2 | 7.0 | 97.4 | 0.42 |
| PC1 in HPCA | 85.7 | 89.5 | 5.14 |
| PC2 in HPCA | 13.0 | 98.7 | 0.78 |

**Supplementary Figure 1.** The variable loadings on first (HPC1) and second (HPC2) principal components in HPCA model.

Meaning of symbols: 1 – principal component from the block X1, 2 – APOPT-BJ (apoptosis of BJ), 3 – APOPT-HEPG2 (apoptosis of HepG2), 4 – APOPT-SW480 (apoptosis of SW480), 5 – sulforaphane, 6 – sinapic acid, 7 – first principal component from the block X2, 8 – second principal component from the block X2
